# Innovative Technique for Wound Management in Combat Zones and Low-Resource Settings: A Mission-Oriented Project for Treating Traumatic Wounds

**DOI:** 10.1093/milmed/usaf250

**Published:** 2025-09-16

**Authors:** Susan St. John, Jonathan Saxe

**Affiliations:** Clinical Development and Education Department, Altrazeal Life Sciences Inc., Addison, TX 75001, United States; Clinical Development and Education Department, Altrazeal Life Sciences Inc., Addison, TX 75001, United States; General Surgery, Surgical Critical Care-Trauma, Ascension St Vincent’s Hospital, Indianapolis, IN 46260, United States

## Abstract

**Introduction:**

Caregivers encounter significant challenges when managing traumatic wounds, especially in resource-limited settings. Frequent dressing changes consume substantial resources, worsen complications such as pain and infection, and contribute to prolonged healing times. Despite advancements in wound care, these issues persist globally, highlighting the urgent need for innovative solutions. This study evaluated the efficacy of an extended-wear transforming powder dressing (TPD) for treating traumatic wounds in a low-resource setting.

**Materials and Methods:**

Altrazeal Life Sciences Inc., in collaboration with the Austrian Development Agency, led a mission-oriented humanitarian project to treat as many patients as possible over a one-year period in resource-constrained public hospitals across India. The project focused on implementing TPD in overburdened facilities to enhance wound healing outcomes and improve resource efficiency in real-world, low-resource settings. After receiving an Institutional Review Board exemption, the study team extracted deidentified project data for a subset of all patients with traumatic wounds (*n* = 39) to evaluate the efficacy of TPD.

**Results:**

All patients who completed at least 2 visits were included in the final analysis (*n* = 23). The cohort consisted of 70% males, with a median age of 40 years (range: 8-82), a median wound duration of 30 days (range: 5-120), and a median wound area of 51.3 cm^2^ (range: 7-600). All patients demonstrated marked clinical improvement, regardless of wound etiology, initial condition, or duration, achieving a median wound area reduction of 51% over a median of 16 days of TPD treatment. Thirty-nine percent of patients healed or were discharged, achieving a 79% median reduction in wound area with a median treatment duration of 31 days. The remaining 61% successfully underwent skin grafting, with a median wound area reduction of 24% after 14 days of TPD treatment. Patients treated with TPD required a median of 1.8 dressing changes per week, compared to 7 with standard care before conversion, resulting in a 75% reduction in dressing change frequency.

**Conclusions:**

Patients treated with TPD showed marked wound area reduction in all trauma wounds with significant overall reduction in dressing change frequency and required time and materials relative to standard of care.

## INTRODUCTION

Trauma injuries from combat or civilian activities are the number 1 cause of death for individuals under 45 years old and rank fourth overall. Each year, these injuries claim over 5.8 million lives, accounting for more than 10% of global deaths.^1^ Dysregulated immune responses frequently accompany trauma, causing severe complications such as delayed wound healing, chronic inflammation, immunosuppression, infection, sepsis, and multi-organ dysfunction. These complications directly drive higher morbidity and mortality rates.^2^

Wound healing plays a crucial role in recovering from traumatic injuries because it directly enables the body to restore damaged tissues and prevent complications. The body repairs damaged skin, muscle, and connective tissues to restore physical function and reduce pain or disability. Open wounds increase the risk of infections or severe conditions like sepsis. Effective healing resolves the inflammatory phase and prevents persistent inflammation, which impairs tissue repair and contributes to chronic complications. Poor wound healing prolongs immune activation and drains resources the body needs to recover from other trauma-related injuries, such as internal organ damage or systemic immune challenges. Chronic wounds that develop from acute injuries further strain medical resources and delay a patient’s return to duty.

Healthcare providers continue to face significant global health challenges in achieving optimal wound management, driving the need for innovative solutions to address the complexity of healing and its associated complications, such as infections, bleeding, and pain. The body progresses through 3 key phases in wound healing: inflammation, proliferation, and remodeling, making it a dynamic and intricate process.^3^ For wounds to heal optimally, care providers must maintain a moist, adequately oxygenated environment that protects against bacterial contamination.^4^ Currently available dressings, however, fail to effectively prevent wound complications, especially on the battlefield or in resource-limited settings. Conventional dressing materials promote bio-burden growth and often trigger a foreign body response when left on wounds for extended periods. Caregivers must frequently change these dressings to manage exudate and prevent infection, which increases patient pain and consumes significant medical resources. In resource-limited settings, shortages of healthcare personnel and materials further complicate these frequent interventions. Although advanced therapies like skin grafts and negative pressure wound therapy effectively manage complex wounds, their high costs, need for specialized skills, and lack of infrastructure often render them inaccessible in conflict zones and resource-limited environments.

## BACKGROUND

An ideal dressing should promote rapid healing at an affordable cost, simplify the care process for providers, and reduce the overall burden on patients and their caregivers. It must offer a multidimensional treatment approach to address the risks of infection, blood loss, pain, and aggravated systemic immune responses associated with traumatic injuries. In this context, we conducted a project to evaluate the efficacy of a transforming powder dressing (TPD) in treating traumatic wounds in a resource-limited setting.

Manufactured and distributed by Altrazeal Life Sciences Inc., USA (ALSI), TPD is a commercially available medical device that has been introduced and adopted in several prominent civilian and Veteran Affairs facilities in the United States, overseas, and in combat hospitals in Ukraine.

TPD has the potential to offer a “smart” and comprehensive dressing system capable of sustained drug delivery, serving as a future platform for the development of customized solutions for all stages of wound healing. Several Phase IV clinical studies are currently comparing TPD to the standard of care (SOC), while pre-clinical studies are exploring new therapeutic combinations of TPD. These efforts are supported by funding from Department of Defense research programs.

**Figure 1.**
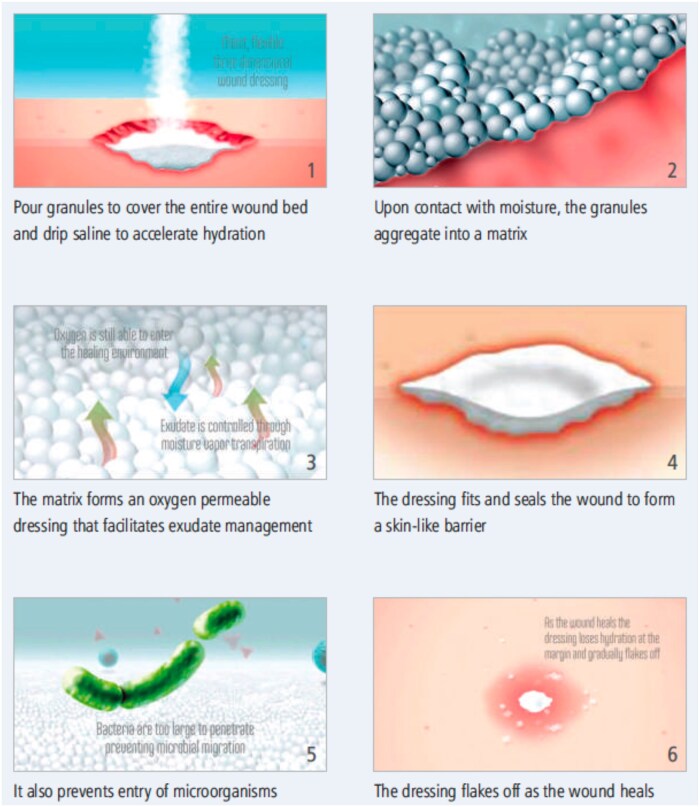
Transforming powder dressing mode of action.

Easy to use with a prolonged wear time of up to 30 days, TPD provides an ideal temporizing cover that can be self-administered or deployed by any caretaker at the point of injury and throughout the continuum of care. Composed primarily of 2 bio-compatible, methacrylate-based hydrogel polymers, TPD is light, easily transportable, and shelf-stable for 4 years. The granules instantly aggregate upon hydration to form a moist, flexible, oxygen permeable barrier that releases excess exudate through vapor transpiration while shielding the wound from bacteria. Simple secondary dressings may be used in cases with high exudate or high friction. Transforming powder dressing can be added from time to time in highly exudative wounds without requiring a full dressing change. Transforming powder dressing dries and flakes off as the wound heals. If required, the nonadhesive TPD can be easily removed atraumatically by moistening with saline and lifting off with forceps (Figure 1).

Clinical and non-clinical studies deliver essential baseline data on TPD’s effectiveness in treating traumatic wounds in low-resource settings. These studies demonstrate TPD’s multifunctional capabilities, establishing it as an ideal wound care covering for early intervention and continued use throughout the continuum of care.

In a porcine full-thickness wound model, TPD quickly aggregated and mimicked a human clot. The dressing contracted and moved upward within the wound bed, promoting granulation tissue formation and supporting robust fibroblast growth (days 2 and 7) and keratinocyte migration (day 14). This occurred alongside normal pro-inflammatory TNFα levels and without requiring any dressing changes. Over the 28-day period, the wounds showed no signs of TPD rejection.^5^

In a porcine study funded by the U.S. Navy Advanced Medical Development Command and conducted in collaboration with the U.S. Army Institute of Surgical Research, researchers tested TPD against 2 standard-of-care solutions (silver-based dressings and gauze) using a 28-day deep partial-thickness burn wound model. The results showed that TPD adhered to the wound bed upon application and flaked off as the wounds healed (days 7 and 14). Histological analysis of day 21 biopsy sections confirmed complete re-epithelialization in TPD-treated wounds, while wounds treated with gauze and silver-based dressings still had open areas. Additionally, TPD-treated wounds displayed a more organized collagen structure, higher Vimentin levels, and faster resolution of inflammatory cell infiltration in the dermal layer compared with the control wounds.^6^

*In vitro* testing demonstrates that TPD acts as an impermeable barrier to bacteria and may inhibit bacterial growth and quorum sensing. Researchers conducted an *in vitro* bacterium micro-barrier study using 3 bacterial strains (*Staphylococcus aureus*, *Pseudomonas aeruginosa*, and *Enterococcus faecalis*) incubated to a concentration of 10^5^ units/microliter.^7^ They applied each strain to either TPD or a control (gauze) in petri dishes and assessed the underlying agar substrates after 28 days of incubation. TPD prevented bacterial growth and provided a clear bacterial barrier. Early clinical evidence supports TPD’s ability to protect wounds from bacterial invasion; in a pilot randomized controlled trial, none of the wounds treated with TPD without antimicrobials became infected.^8^

Clinical evaluations of both chronic and acute wounds reveal that numerous patients experience accelerated healing, reduced dressing change frequency, and reported enhanced comfort, including less pain and reduced use of analgesics. A randomized, prospective, non-inferiority, between-patient pilot study compared split-thickness skin graft donor sites of burn patients treated with TPD to those treated with a silver-carboxymethylcellulose dressing. The results showed that patients treated with TPD reported significantly less pain at 3 time intervals (2-5 days, 6-10 days, and 11-15 days; *P* < .001). Patients also reported greater comfort with TPD.^8^ In a prospective case series involving 9 partial-thickness burns (TBSA (total body surface area) 2-12%), all subjects reported a 67-86% reduction in pain and achieved healing with a single TPD application in an average of 12.1 days (range: 9-16 days).^9^ A splinted wound is a less painful wound and demonstrates less disruption or interference with wound healing. In a retrospective analysis of data from 10 patients (mean age of 42) with complex, full thickness, traumatic wounds with elements of exposed bone, tendons, and muscle, 6 patients achieved complete healing with TPD without requiring grafting. The mean healing time was 23 days with 3 TPD top-offs (approximately once per week).^10^ Four patients received successful grafting treatments once sufficient granulation had been achieved with TPD treatment for an average of 33 days and TPD applications every 6 days on average.

Various chronic wound studies demonstrate the promising potential of TPD as a universal wound care solution beyond acute wounds. In a randomized controlled study (*n* = 60) with chronic venous ulcers (average wound duration > 30 months), the TPD arm achieved a higher healing rate (87.6% vs. 40.0%, *P* < .001), faster healing (16.7 days vs. 37.0 days, *P* < .001) and required significantly fewer dressing changes (23.9 in the TPD cohort versus 196 in SOC (*P* < .001)) relative to SOC over a 24-week period. Mean pain score and percentage of patients on analgesics were lower for TPD patients (VAS score of 1.4 vs. 2.9 with SOC (*P* < 0.001); 2% of TPD patients were on analgesics vs. 11% for SOC (*P* = 0.005)).^11^ In a retrospective case series (*n* = 21) with non-healing, Stage 2–4 pressure injuries (PrIs), all patients (mean age of 50) experienced successful and expedited wound closure, with an average healing period of 52 days and 4 dressing changes (∼14 days between dressing changes).^12^ On average, Stage 4 PrIs closed in 87 days with 6 dressing changes, Stage 3 PrIs closed in 41 days with 4 changes, and Stage 2 PrIs healed in 13 days with 1 application. Patients with painful wounds experienced significant pain reduction; pain scores declined from 8-9/10 to 1-2/10. A retrospective evaluation of 17 patients (mean age of 58) with non-healing, Wagner Grade 2-3 diabetic foot ulcers with mean duration of 9.5 months, all patients displayed accelerated wound closure and avoided amputations.^13^ The mean number of dressing changes was 6, and the time to heal was 46 days.

Available preclinical and clinical data position TPD as a viable alternative for treatment of traumatic wounds in low resource settings.

## METHOD

The study is a post-marketing review of de-identified secondary case data collected from patients with traumatic wounds treated during a humanitarian project conducted by ALSI in collaboration with the Austrian Development Agency (ADA). During the COVID-19 pandemic, ALSI and ADA led a mission-oriented humanitarian project to support patients and care providers in a low-resource setting. The project aimed to treat as many patients as possible over a 1-year period by implementing TPD in resource-constrained public hospitals across India.

As part of the project, ALSI provided TPD free of cost to medical facilities without any restrictions on product usage and trained hospital staff on its effective application across all wound types. Physicians applied TPD at their own discretion to patients with diverse wound types, including surgical, traumatic, and chronic wounds. No specific criteria for wound selection were applied as the common goal of the humanitarian project was to treat as many patients as possible while minimizing patient encounters during a challenging COVID-19 environment. A total of 195 patients were treated across 37 hospitals. Clinical staff recorded data as part of their routine care visits. All patients were transitioned from standard care (SOC) to TPD treatment, continuing with TPD until their wounds healed, were grafted, or they were discharged. SOC wound management modalities utilized before TPD include conventional dressings including gauze wraps, pads, or paraffin gauze.

For purposes of the post-marketing review, de-identified data related to a subset of patients with traumatic wounds were extracted from site records using data collection forms and evaluated retrospectively. The study was reviewed by the Ascension St Vincent’s Hospital (Indianapolis, IN) Institutional Review Board (IRB) and an exemption determination was obtained before initiating the review. The exemption was based on the use of de-identified secondary case data from patients receiving commercially available TPD under standard of care conditions through humanitarian efforts in a non-research setting.

The extracted dataset included all patients with traumatic wounds treated during the ADA project (*n* = 39). To ensure broad applicability, the study imposed no restrictions on age, wound location, or etiology for eligibility.

The study aimed to evaluate the impact of TPD (treatment intervention) on wound healing, dressing change frequency, and related adverse events. There was no control group. Data for dressing change frequency were compared to data available regarding frequency of dressing changes with SOC before patient transition to TPD.

The study analyzed patient demographics, wound dimensions, the number and frequency of dressing changes, and time to healing, grafting, or discharge.

## RESULTS

### Demographics

All patients who completed at least 2 visits were included in the final analysis (*n* = 23). The COVID-19 pandemic led to the loss of follow-up for 16 patients. The study cohort included 70% male participants, with a median age of 40 years (range: 8-82 years). This broad age range reflects the typical population of patients seen at public hospitals in India, where the study was conducted. The study team observed a median wound area of 51.3 cm^2^ (range: 7-600 cm^2^). Wound age also varied with a median duration of 30 days (range: 5-120 days) encompassing both acute and chronic conditions (Table 1). The population included full thickness wounds from various etiologies (from skin tears to more severe accident-related trauma) in challenging locations, including the pelvis, scrotum, legs, foot, back, neck, chest, hips, and ankles. The diverse sample set enabled an evaluation of TPD across a broad range of wound etiologies, chronicity, and sizes.

**Table 1.** Baseline Characteristics and Outcomes by Study Cohort^a^

	Total population analyzed (*n* = 23)	Patients healed/discharged (*n* = 9)	**Patients referred for grafting (*n* = 14)** ^a^
Median age in years (range)	40 (8-82)	37 (8-82)	41 (25-68)
Male/female, *n*	16/9	6/3	10/4
Median duration of wounds before TPD (range)	30 (5-120)	30 (30-60)	35 (5-120)
Median baseline wound area (cm^2^) (range)	51 (7-600)	57 (7-595)	50 (15-600)
Median dressing changes per week with SOC	7	7	7
**Median TPD treatment duration (Days)**	**16**	**31**	**14**
**% Reduction in median wound area over TPD treatment period**	**51%**	**79%**	**24%**
Median dressing changes per week with TPD	1.8	1.4	2.0
**% Reduction in median dressing changes per week with TPD**	**75%**	**80%**	**71%**

a Wound area reduction excludes data from 1 burn patient with multiple burns of 15% TBSA for which wound dimensions were not recorded.

### Wound Healing

**Figure 2.**
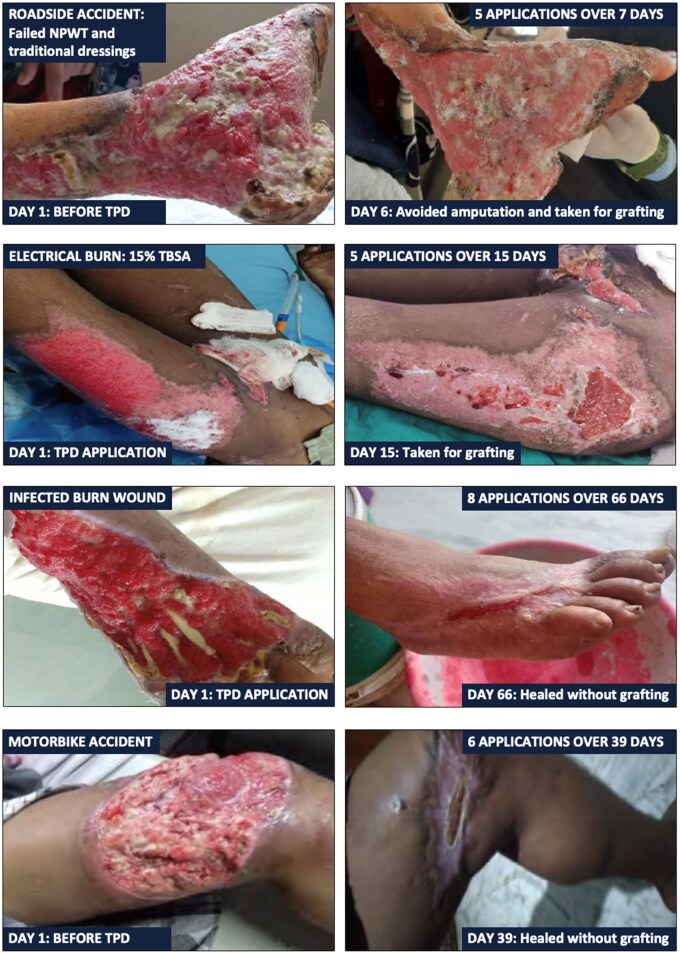
Illustrative Cases*(*TPD applications include “top-offs” or addition of TPD without primary dressing changes)

All patients demonstrated marked clinical improvement, regardless of wound etiology, initial condition, or duration, achieving a median wound area reduction of 51% over a median of 16 days of TPD treatment. Thirty-nine percent of patients (*n* = 9, including 5 infected wounds) healed or were discharged, achieving a 79% median reduction in wound area with a median treatment duration of 31 days. Additionally, clinicians performed successful skin grafting procedures on 61% of patients (*n* = 14). Before grafting, wounds showed a 24% median wound area reduction over after 14 days of TPD treatment (Figure 2).

### Dressing Change Efficiency

**Figure 3.**
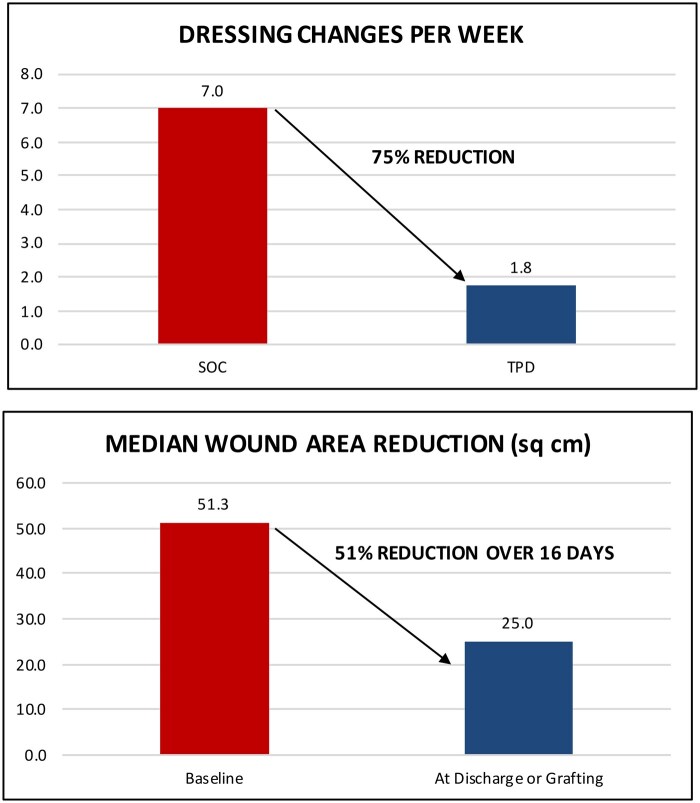
Summary results. Wound area reduction excludes data from 1 burn patient with multiple burns of 15% TBSA for which wound dimensions were not recorded.

Before receiving TPD treatment, clinicians treated all patients with SOC dressings, which required daily or every-other-day changes using gauze wraps, pads, or paraffin gauze. After switching to TPD, healthcare providers applied or topped off TPD approximately once every 4 days. Patients treated with TPD required a median of 1.8 dressing changes per week, compared to 7 with standard care before conversion, resulting in a 75% reduction in dressing change frequency (Figure 3).

### Resource Utilization

Clinicians reduced dressing change frequency, which lowered material usage, minimized medical supply consumption, and decreased associated waste and costs.

### Adverse Events

No TPD-related adverse events were reported throughout the study. No secondary infections were reported.

## DISCUSSION

The COVID-19 pandemic and associated lockdowns significantly reduced the study’s sample size, leading to 16 patients being lost to follow-up. The study was also limited by the scope of a post-marketing review that did not include randomized treatment and control groups. Since the extracted data was obtained from routine data parameters collected by hospitals, data regarding the long-term effects on scarring and mobility were not available. Despite the study limitations, the observed results demonstrate that TPD offers a promising solution for managing traumatic wounds, especially in resource-constrained environments. Transforming powder dressing enhanced wound healing to full closure or prepared wounds for grafting while reducing the frequency of dressing changes across a wide range of wound locations. These findings position TPD as a versatile and comprehensive wound care intervention for traumatic wounds. Its efficient use of resources, absence of product-related adverse events, and ability to improve outcomes make it a valuable addition to the wound care toolkit.

Building on these promising findings, larger, randomized, controlled DoD-funded studies are actively comparing TPD to SOC in the treatment of acute wounds (burns) and chronic wounds (diabetic foot ulcers, pressure injuries), paving the way for broader adoption of this innovative technology.

## CONCLUSION

The collaboration between ALSI and the ADA exemplifies the power of public-private partnerships in tackling global health challenges. The project demonstrated that TPD provides an effective and resource-efficient solution for managing traumatic wounds in underserved settings. By leveraging its extended-wear properties, TPD significantly reduced dressing change frequency, optimized resource utilization, and achieved marked improvements in wound healing outcomes.

When combined with findings from other studies conducted in both resource-limited and advanced healthcare settings, these data underscore TPD’s broad applicability and versatility. Its ability to manage diverse wound types, including acute and chronic wounds, positions TPD as a practical and adaptable solution that meets the varying needs of patients across all care settings.

Ongoing research continues to expand TPD’s potential. With funding support from U.S. Department of Defense research programs, larger randomized controlled trials are underway, alongside studies exploring TPD combinations with antibiotics and other therapeutic agents. These efforts highlight TPD’s role in advancing wound care innovation.

Through continued collaboration and innovation, TPD has the potential to transform global wound care standards. Its clinical effectiveness, ease of use, and adaptability make it particularly valuable in regions with limited medical resources, offering hope for improved care and outcomes for underserved populations worldwide.

## Data Availability

The corresponding author will share the data underlying this article upon reasonable request.

## INSTITUTIONAL CLEARANCE

Institutional clearance does not apply.
